# Acute mountain sickness induced diabetic ketoacidosis managed with hemodialysis: A case report

**DOI:** 10.1016/j.amsu.2020.06.012

**Published:** 2020-06-13

**Authors:** Kamal Pandit, Sushil Khanal, Samaj Adhikari, Subhash Prasad Acharya

**Affiliations:** a Department of Critical Care, Grande International Hospital, Kathmandu, Nepal; b Institute of Medicine, Maharajgunj, Kathmandu, Nepal; c Professor, Department of Anaesthesiology, Institute of Medicine, Maharajgunj, Kathmandu, Nepal

**Keywords:** Diabetes ketoacidosis, Acute mountain sickness, High altitude, Hemodialysis

## Abstract

**Introduction:**

The risk of developing ketoacidosis in patients with type 1 diabetes at high altitude is high. Anorexia associated with acute mountain sickness, dehydration and additional exercise associated with climbing exacerbates the generation of ketones and the development of ketoacidosis.

**Case presentation:**

A 33-year-old gentleman with known history of uncontrolled type 1 diabetes mellitus trekked to Everest Base Camp at an altitude of 3440 m and became unwell. He developed altered sensorium and shortness of breath. He ingested eight tablets of acetazolamide (250 mg each) to address these symptoms. Upon presentation to emergency, he was diagnosed with severe diabetes ketoacidosis (DKA) with shock. Resuscitation was started with fluid, insulin, vasopressors and mechanical ventilation. Despite adequate fluid resuscitation, insulin, bicarbonates and other supportive measures, his acidosis and shock persisted and then managed with hemodialysis. After the first session of hemodialysis, improvement in acidosis and shock was noted. He was successfully extubated and later discharged.

**Discussion:**

In this case report, DKA due to acute mountain sickness was complicated by acetazolamide use and noncompliance to his regular insulin intake. There is no proper guideline regarding the role of renal replacement therapy in management of DKA. However, evidence of hemodialysis in DKA is limited to few case reports. Improvement seen in our patient after dialysis is related to dialyzable nature of acetazolamide.

**Conclusion:**

We present a case of a severe DKA potentially precipitated by acute mountain sickness, use of acetazolamide, noncompliance to his regular insulin intake and managed with hemodialysis in addition to conventional treatment for DKA.

## Highlights

• Metabolic decompensation that occur with high altitude increases the generation of ketones and the development of ketoacidosis.
• Incorporating renal replacement therapy in severe refractory acidosis in DKA management will reduce the morbidity and mortality in patient with DKA.
• The timely intervention of dialysis in severe refractory acidosis has a good outcome.

## 1 Introduction

Diabetes ketoacidosis (DKA) is a complex medical emergency characterized by hyperglycemia, metabolic acidosis and ketosis. Various stressful conditions that decompensate metabolic demand lead to DKA [1,2]. The association of DKA with different precipitating events has been mentioned in literatures, but there are few reports mentioning the acute mountain sickness triggering this life-threatening condition. Different pathophysiological changes that occur at high altitude can lead to DKA in diabetes patients [3]. The major elements of treatment of DKA include fluid resuscitation, insulin therapy, electrolyte replacement and optimization of potential precipitating factors. Rarely, dialysis has been used as a treatment modality for refractory acidosis associated with DKA [[4], [5], [6], [7]]. Here we present a rare case of acute mountain sickness induced DKA with refractory acidosis managed with hemodialysis.

### 1.1 Case summary

A 33-year-old gentleman with known history of uncontrolled type 1 diabetes mellitus trekked to Everest Base Camp for 4 days at an altitude of 3440 m. He became unwell and developed altered sensorium and shortness of breath. He had missed his insulin doses for last 2 days. He ingested 8 tablets of acetazolamide (250 mg each) over 24 hours for his shortness of breath while trekking. He also had nausea, headache, loss of appetite and palpitation. He was then taken to a nearby primary center at low altitude by his friends where his primary supportive care was done with oxygenation and intravenous fluid. He was then referred to our hospital in Kathmandu via helicopter transport.

On presentation to our Emergency he was drowsy and his Glasgow Coma Scale (GCS) was E2V3M5, breathing was labored with respiratory rate of 34 per minutes, SPO2 was 86% with supplemental oxygen, blood pressure was 90/70 mm of Hg, heart rate of 128/min, and temperature of 37 °C. The patient was dehydrated. However, there was no pallor, icterus, palpable lymph node or peripheral edema. The jugular venous pressure was not raised. There were no signs of microvascular and macrovascular complications.

### 1.2 Investigations

Laboratory investigation revealed random blood sugar of 433 mg/dl with HbA1c 16.9%. His urine acetone was positive. Blood gas analysis at the time of admission showed pH of 6.89, bicarbonate of 2.0 meq/dl, anion gap of 44 and lactate of 1.4 mmol/l. His serum sodium was 126 mmol/l, potassium 3 mmol/l, chloride 80 mmol/l, blood urea 49 mg/dl, serum creatinine 0.9 mg/dl. His blood ketone level was 2.2 mmol/l. His hemoglobin was 17.4 g/dl, total leucocyte counts of 36,850/mm^3^ and platelet count 218,000/mm^3^. On chest auscultation crackles were present on bilateral chest. On chest radiography, there was patchy infiltration in the bilateral lung fields. The EKG was normal. CT head was done for low GCS and drowsiness and the findings were normal.

### 1.3 Treatment, outcomes and follow ups

Fluid resuscitation was started with multiple boluses of normal saline and continued as per requirement. Acetazolamide was stopped and dexamethasone was started. Potassium replacement was also done for hypokalemia. In addition, infusion of regular insulin at 6–12 U/h with 5% dextrose was initiated. Despite ample fluid boluses the patient was hypotensive thus requiring multiple vasopressors (noradrenaline, vasopressin and adrenaline). He was also given intravenous sodium bicarbonate infusion in the initial 12 hours due to severe metabolic acidosis. He was intubated because of persistent shock, decreasing consciousness level and hemodynamic instability.

He received about 6 L of fluid in first 6 hours with urine output of about 1.5 L. His blood glucose level was normal. However, his metabolic acidosis and shock were persistent as the blood gas analysis showed pH of 6.99, bicarbonate of 5.5 meq/dl, anion gap of 24.5 and lactate of 1.5mmlo/l. Then he was started on hemodialysis using Fresenius 4008B, Fresenius medical care at the following settings: blood flow, 100 mL/min; duration,4hr; anticoagulation, unfractionated heparin 1000 IU. After 12 hours of single session of hemodialysis, his arterial blood pH improved to 7.35 with bicarbonate of 18.5 meq/dl. Furthermore after 36 hours of hemodialysis his arterial blood showed pH of 7.45 with bicarbonate of 27.6 meq/dl. The improvement on pH and HCO3- has been summarized in Table 1. The total amount of intravenous insulin he received during his treatment was 80 IU. Over the course of his stay, vasopressors were tapered off and his GCS improved to E4M6Vt and he was subsequently extubated on day six of admission. He was shifted to step down unit on day eight with improvement in his metabolic and hemodynamic parameter. Finally, he was discharged home on day fourteen of illness. After a week of follow up he was completely asymptomatic.

**Table 1:** Improvement in pH and HCO3-with hemodialysis(HD).

Time since admission	pH	HCO3-	Na+/Cl-	K^+^	Anion Gap
0 h	6.89	2.0	126/80	3.0	44.0
6 h	6.99	5.5	151/121	2.9	24.5
12 h (start of HD)	7.15	6.7	154/120	2.2	27.3
24 h (12 h after HD)	7.35	18.5	153/119	3.6	15.5
36 h (24 h after HD)	7.42	25.2	146/109	3.4	11.8
48 h (36 h after HD)	7.45	27.6	146/108	3.7	10.4
72 h (48 h after HD)	7.47	27.7	151/111	3.7	12.3

## 2 Discussion

DKA may be precipitated by multiple factors impacting glucose regulation. In this case report we believe that, DKA manifested because of his trek to high altitude causing acute mountain sickness and further complicated by acetazolamide use and noncompliance to his regular insulin intake. Studies have shown patients missing the insulin dose and underlying infections mainly predispose to DKA [8,9].

Complications of altitude did not occur with greater frequency in the diabetic climbers but certain metabolic decompensation that occurs with high altitude increases the risk of DKA. Anorexia, dehydration and additional exercise associated with climbing during high altitude exacerbate the generation of ketones and the development of ketoacidosis [10].

Our patient did not show improvement in his metabolic acidosis despite adequate fluid resuscitation, insulin and sodium bicarbonate. The role of sodium bicarbonate as a therapy for diabetic ketoacidosis (DKA) is controversial. The Systematic review by Chua et al. concluded that there is no role of sodium bicarbonate in DKA management [11]. However, American Diabetes Association recommends the administration of 100 mmol sodium bicarbonate in 400 ml sterile water with 20 meq of KCl to patients with a pH of less than 6.90 until the pH rises above 7.00 [12]. We decided for hemodialysis in our patient for severe refractory metabolic acidosis and hemodynamic instability. He showed improvement in metabolic acidosis and shock after hemodialysis.

There is no proper guideline regarding the role of renal replacement therapy in management of DKA. However, evidence of hemodialysis in DKA is limited to few case reports [4,13]. Miller and Ahmad reported DKA in high altitude managed with conventional treatment measures [14,15]. Additionally, Moore et al. reported ketoacidosis in high altitude managed with conventional treatment measures but they did not mention how they corrected persistent acidosis in one of the mountaineers [10].

Acetazolamide use in our patient might have helped precipitating DKA. Acetazolamide inhibits the carbonic anhydrase enzyme that results in reduction of hydrogen ion in the renal tubules and thereby blocks reabsorption of bicarbonate in the renal tubules which leads to metabolic acidosis [16]. The low level of bicarbonate i.e. 2.0mmol/l in this patient is due to impaired reabsorption and increased washout of bicarbonate from the renal tubules due to acetazolamide. The acetazolamide level peaks at 2 hours of its oral intake and has estimated half-life of 4–8 hours [17]. Acetazolamide might have also led to persistent acidosis in our patient despite normal blood glucose and electrolytes level. This has been observed in previous study [18]. A significant amount of acetazolamide (about 30%) can be removed by 4 hours of single session of hemodialysis despite its high protein binding nature [19]. Improvement seen in our patient after dialysis could also be related to dialyzable nature of acetazolamide.

An activity at high altitude is associated with increased energy demand that may lead to dysregulation of glucose balance unless adjustment in medication is performed. There will be high risk of ketone production if energy expenditure is not matched by calorie intake [3,10]. Although there are potential medical risks of high altitude to diabetics, individuals with well controlled diabetes can do activities at high altitude with vigilance, well preparation and knowledge about the risks.

In this case report, we emphasized on severe diabetic ketoacidosis precipitated by high altitude with other factors and managed with hemodialysis in addition to other conventional treatment measures. Knowledge about the pathophysiology of DKA at high altitude and incorporating renal replacement therapy in severe refractory acidosis in DKA management will reduce the morbidity and mortality in this patient type in the future.

### 2.1 Learning points

1. The risk of developing ketoacidosis in patient with type 1 diabetes at high altitude is considerably high as metabolic decompensation that occur with high altitude increases the generation of ketones and the development of ketoacidosis.

2. Knowledge about the pathophysiology of DKA at high altitude and incorporating renal replacement therapy in severe refractory acidosis in DKA management will reduce the morbidity and mortality in patient with DKA.

3. Although dialysis has been rarely used as a treatment modality for refractory acidosis associated with DKA, the timely intervention of dialysis in such conditions has a good outcome.

## Ethical approval

Research studies involving patients require ethical approval. Please state whether approval has been given, name the relevant ethics committee and the state the reference number for their judgement.

This case report was conducted in compliance with ethical standards. Informed written consent has been obtained and all identifying information is omitted.

The following information is required for submission. Please note that failure to respond to these questions/statements will mean your submission will be returned. If you have nothing to declare in any of these categories then this should be stated.

## Consent

Studies on patients or volunteers require ethics committee approval and fully informed written consent which should be documented in the paper.

Authors must obtain written and signed consent to publish a case report from the patient (or, where applicable, the patient's guardian or next of kin) prior to submission. We ask Authors to confirm as part of the submission process that such consent has been obtained, and the manuscript must include a statement to this effect in a consent section at the end of the manuscript, as follows: “Written informed consent was obtained from the patient for publication of this case report and accompanying images. A copy of the written consent is available for review by the Editor-in-Chief of this journal on request”.

Patients have a right to privacy. Patients’ and volunteers' names, initials, or hospital numbers should not be used. Images of patients or volunteers should not be used unless the information is essential for scientific purposes and explicit permission has been given as part of the consent. If such consent is made subject to any conditions, the Editor in Chief must be made aware of all such conditions.

Even where consent has been given, identifying details should be omitted if they are not essential. If identifying characteristics are altered to protect anonymity, such as in genetic pedigrees, authors should provide assurance that alterations do not distort scientific meaning and editors should so note.

The informed written consent was taken from patient for the publication of this case report.A copy of the written consent is available for review by the Editor in chief of this journal on request.

## Author contribution

Please specify the contribution of each author to the paper, e.g. study concept or design, data collection, data analysis or interpretation, writing the paper, others, who have contributed in other ways should be listed as contributors.

1. Kamal Pandit took relevant history, clinical examination, collected relevant investigations of the patient and wrote the report. And he was directly involved in patient's care during his stay in ICU.
2. Sushil Khanal also wrote the report and revised it with relevant references. And he was directly involved in patient's care during his stay in ICU.
3. Subhash Prasad Acharya provided support and mentorship for development, writing and revision of this case report. And he was directly involved in patient's care during his stay in ICU.
4. Samaj Adhikari worked for literature review and revision of the case report into its final version. He was not directly involved in the patient's care.

## Registration of research studies

In accordance with the Declaration of Helsinki 2013, all research involving human participants has to be registered in a publicly accessible database. Please enter the name of the registry and the unique identifying number (UIN) of your study.

You can register any type of research at http://www.researchregistry.com to obtain your UIN if you have not already registered. This is mandatory for human studies only. Trials and certain observational research can also be registered elsewhere such as: ClinicalTrials.gov or ISRCTN or numerous other registries.

1. Name of the registry: Not applicable
2. Unique Identifying number or registration ID:
3. Hyperlink to your specific registration (must be publicly accessible and will be checked):

## Please state any conflicts of interest

All authors must disclose any financial and personal relationships with other people or organisations that could inappropriately influence (bias) their work. Examples of potential conflicts of interest include employment, consultancies, stock ownership, honoraria, paid expert testimony, patent applications/registrations, and grants or other funding.

There is no any conflicts of interest.

## Please state any sources of funding for your research

All sources of funding should be declared as an acknowledgement at the end of the text. Authors should declare the role of study sponsors, if any, in the collection, analysis and interpretation of data; in the writing of the manuscript; and in the decision to submit the manuscript for publication. If the study sponsors had no such involvement, the authors should so state.

This study has not received any funding.

## Provenance and peer review

Not commissioned, externally peer reviewed.

Annals of Medicine and Surgery.

## Guarantor

The Guarantor is the one or more people who accept full responsibility for the work and/or the conduct of the study, had access to the data, and controlled the decision to publish.

Kamal Pandit.He is the first author and corresponding author for this case report.
